# Residual stone fragments: systematic review of definitions, diagnostic standards

**DOI:** 10.1007/s00345-025-05572-x

**Published:** 2025-03-28

**Authors:** O. F. Çavdar, A. Aydin, T. Tokas, A. Tozsin, N. Gadzhiev, M. G. Sönmez, R. Tekeli, G. Ortner, P. Kallidonis, B. Akgül, T. Knoll, G. Bianchi, J. Rassweiler, K. Ahmed, S. Guven

**Affiliations:** 1 Necmettin Erbakan University, Urology, Konya, Türkiye; 2 Faculty of Life Science and Medicine, King’s College London, London, UK; 3 Department of Urology, University General Hospital of Heraklion, University of Crete, Medical School, Heraklion, Greece; 4 Training and Research in Urological Surgery and Technology (T.R.U.S.T.)-Group, Hall in Tirol, Austria; 5 Trakya University School of Medicine, Urology, Edirne, Türkiye; 6 Department of Urology, Saint-Petersburg State University Hospital, Urology, Saint-Petersburg, Russia; 7 Department of Urology, General Hospital Hall I.T, Urology, Tirol, Austria; 8 Department of Urology, University Hospital of Patras, Patras, Greece; 9 Klinikum Sindelfingen-Boeblingen, University Medicine Mannheim, Urology, Mannheim, Germany; 10 Deparment of Urology, Università Degli Studi Di Modena e Reggio Emilia, Urology, Modena, Italy; 11 Department of Urology and Andrology, Danube Private University, Urology, Krems, Austria; 12 Sheikh Khalifa Medical City, Abu Dhabi, United Arab Emirates; 13 Khalifa University, Abu Dhabi, United Arab Emirates

**Keywords:** Extra-corporeal shockwave lithotripsy, Retrograde intrarenal surgery, Percutaneous nephrolithotomy, Residual stone fragments

## Abstract

**Purpose:**

Residual stone fragments (RSFs) remain a determining factor for evaluation of outcome an intervention for management of renal tract stones. However, there is a lack of consensus on size, location, diagnosis and management of RSF. This systematic review aims to assess definitions and diagnostic approaches to RSF across urolithiasis treatment modalities while standardizing their definition and diagnosis through a systematic review, stratifying RSF patients into risk groups, and proposing an approach for management.

**Materials and methods:**

A comprehensive literature search was conducted, using Preferred Reporting Items for Systematic Reviews and Meta-Analyses (PRISMA) guidelines (PROSPERO ID: CRD42024603807). Embase, MEDLINE (PubMed) and Cochrane databases were searched until July 2024. Twentynine studies were included and categorized according to treatment choices i.e. extra-corporeal shockwave lithotripsy (ESWL) (*n* = 12), retrograde intrarenal surgery (RIRS) (*n* = 7), and percutaneous nephrolithotomy (PCNL) (*n* = 10). Each study’s quality was evaluated using the Quadas Scoring System to determine the risk of bias and concerns regarding applicability. We included original studies that systematically defined and proposed approaches for RSF definition and diagnosis. Based on the emerging categories, we proposed a risk stratification model to classify patients accordingly.

**Results:**

RSF definitions varied, with most studies defining RSF as fragments < 4 mm, though thresholds of < 2 mm and < 5 mm were also common. Definitions typically included only asymptomatic fragments without obstruction or infection. Computed tomography (CT) was the imaging modality most selected for diagnosis and was used in 14 studies. The timing of imaging modalities for follow-up was highly heterogeneous. The incidence of RSFs following ESWL has been reported between 21% and 59% across the studies. Among the RIRS studies, RSF rates varied between 20 and 60.5% of patients, and RSFs were observed between 20 and 60% after PCNL. The variability in RSF definitions affects comparability and may impact reintervention rates and treatment outcomes.

**Conclusion:**

This systematic review highlights inconsistencies in defining RSFs, with common thresholds being < 2 mm, < 4 mm, or < 5 mm. CT is noted as the most reliable method for assessing fragment size and location. RSFs over 4 mm, particularly in the lower pole, are associated with higher risks of progression and complications. The review advocates to adopt standardized definitions and imaging protocols to enhance comparability and patient outcomes.

## Introduction

Residual stone fragments (RSFs) following shock wave lithotripsy (ESWL) or endourological procedures such as percutaneous nephrolithotomy (PCNL) and retrograde intrarenal surgery (RIRS) represent a significant challenge in the management of urolithiasis. While traditionally considered an acceptable clinical endpoint, RSFs can lead to adverse outcomes, including stone regrowth, infection, obstruction, and the need for re-intervention, posing a substantial burden on healthcare systems [1–3]. Accurate detection of RSFs relies on imaging modalities like non-contrast computed tomography (NCCT), ultrasonography (US), and radiographs, each with specific strengths and limitations. NCCT is the gold standard due to its superior sensitivity in detecting even small fragments but concerns about cost and radiation exposure remain [4]. Despite technological advancements, such as developing enhanced irrigation and laser systems during endourological procedures to minimize RSFs, challenges persist in standardizing their use and demonstrating clear long-term benefits in reducing fragment-related complications [5].

Achieving a stone-free status is a critical outcome of urolithiasis treatment; however, the lack of standardized definitions for residual fragments complicates clinical management. While the European Association of Urology (EAU) guidelines define “stone-free” status as the absence of fragments detectable on imaging such as CT, kidney-ureter-bladder (KUB) graphs, or the US, there is no consensus on the criteria for RSFs’ clinical significance [6]. Definitions vary widely, with some studies considering RSFs smaller than 4 mm in asymptomatic patients clinically insignificant, while others suggest size and composition are critical factors [7]. These inconsistencies in definition and management and variability in follow-up protocols and imaging modalities highlight the need for a unified approach. Both clinical research and consensus should be built on fundamental principles such as definition, standardization, and proper classification of the matter. Despite the frequent mention of RSFs in studies, a comprehensive and methodologically structured evaluation is required, systematically addressing the definitions, classification, and management of residual fragments in urolithiasis. This systematic review aims to evaluate the current literature regarding RSFs in terms of standardizing the definitions, selecting diagnostic modalities, stratifying RSF patients into risk groups, and proposing an approach for management.

## Materials and methods

### Search strategy

**Fig. 1:**
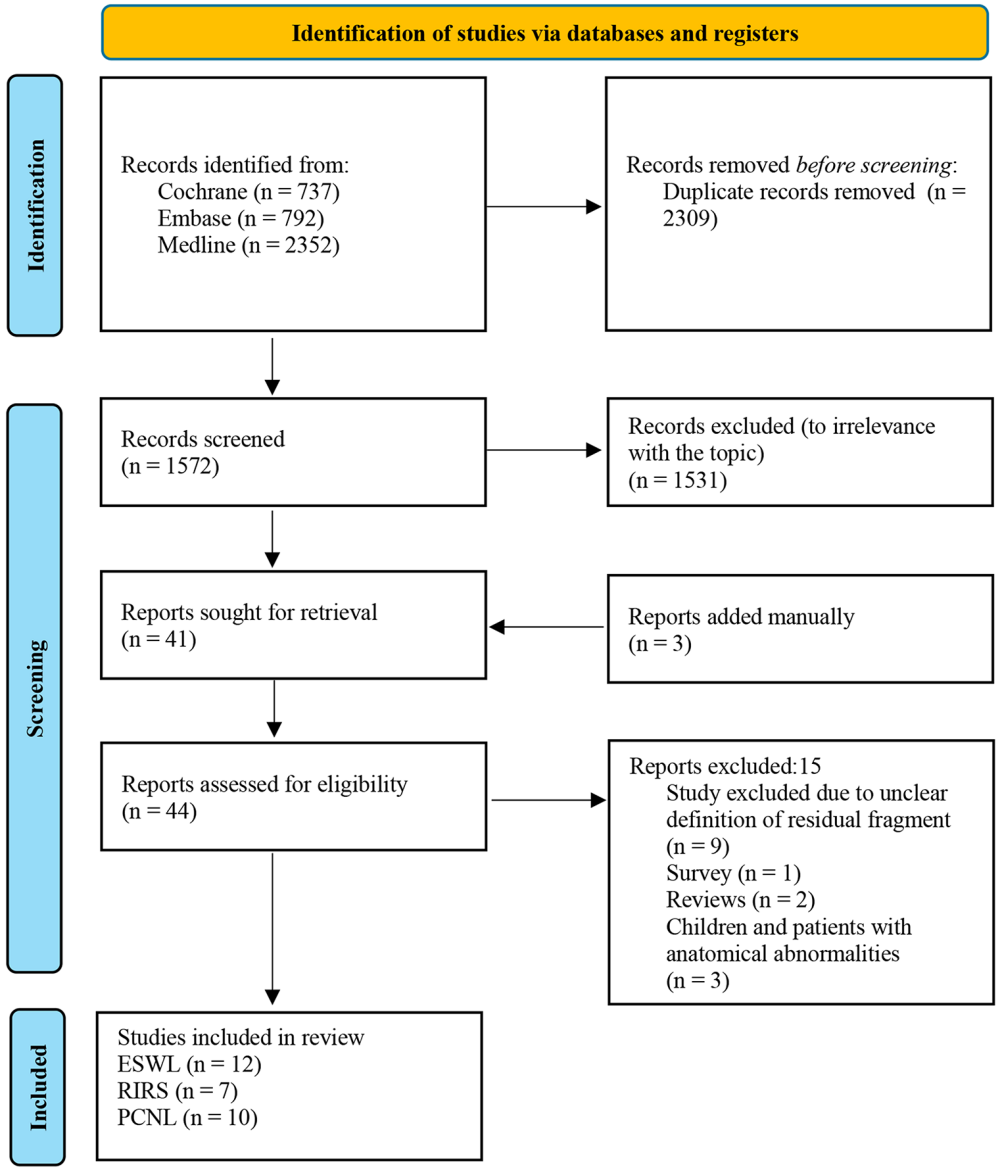
The flowchart of the systematic review according to PRISMA guidelines

This review (PROSPERO ID: CRD42024603807) followed the Preferred Reporting Items for Systematic Reviews and Meta-Analyses (PRISMA) guidelines (Fig. 1). A comprehensive literature search was conducted up to July 2024 with no time restriction. We used a combination of Medical Subject Headings (MeSH) and keywords across the Embase, MEDLINE (PubMed) and Cochrane databases. The analysis focused on articles concerning adults and were published in the English language. The search terms included ‘Stones’, ‘kidney stones’, ‘renal stones’, ‘urolithiasis’, ‘residual fragment(s)’, ‘clinically insignificant residual fragment(s)’, ‘residual stone fragment’ in combination with ‘SWL’, ‘ESWL’, ‘RIRS’, ‘PCNL’, ‘percutaneous nephrolithotomy’, ‘extracorporeal shockwave lithotripsy’, ‘retrograde intrarenal stone surgery’. Boolean operators (AND, OR) were used to refine the search.

### Study selection

Initially, 3881 records were identified. After duplicates were removed (*n* = 2309), 1572 records remained for further screening. The screening process produced 1531 records that were excluded due to irrelevance to the topic by title review and abstract review. A total of 41 reports were searched and three additional reports were added manually. As a result, 44 reports were assessed for eligibility. During full-text assessment, 15 reports were excluded for the following reasons: nine studies were excluded due to unclear definitions of RSFs, one was a survey, two were investigations, and three included children or patients with anatomic abnormalities. In total, 29 studies were included and categorized according to their treatment choice: ESWL (*n* = 12), RIRS (*n* = 7), and PCNL (*n* = 10). Included studies were identified based on the evaluation of full-text content, which had institutional review board (IRB) and ethics committee (EC) approval [8–21]. Each study was assessed by at least two independent reviewers who assessed titles, abstracts, and full-text articles separately. Any disagreement between the two reviewers (O.F.C., A.T.) was resolved through discussion and if consensus could not be reached, a third reviewer (B.A.) made an additional assessment to finalise the decision.

### Data extraction

Data were extracted independently by two investigators. The following general information was recorded: type of intervention, first author and year of publication, study type, patient demographics, size, number, location of the RSFs, features of clinical presentation, type of imaging, Hounsfield Units (HU) of the RSFs, diagnostic method used, time between surgery and imaging of the RSFs, and whether the patient was stone-free or not. The findings were analyzed to provide a comprehensive summary of the current understanding of the topic, highlighting key observations. Given the retrospective nature of most studies and the variations in their designs, the results were synthesized using a narrative approach. We included original studies that systematically defined and proposed approaches for RSF definition and diagnosis. Based on the emerging categories, we proposed a risk stratification model to classify patients accordingly.

### Study qualification

**Fig. 2:**
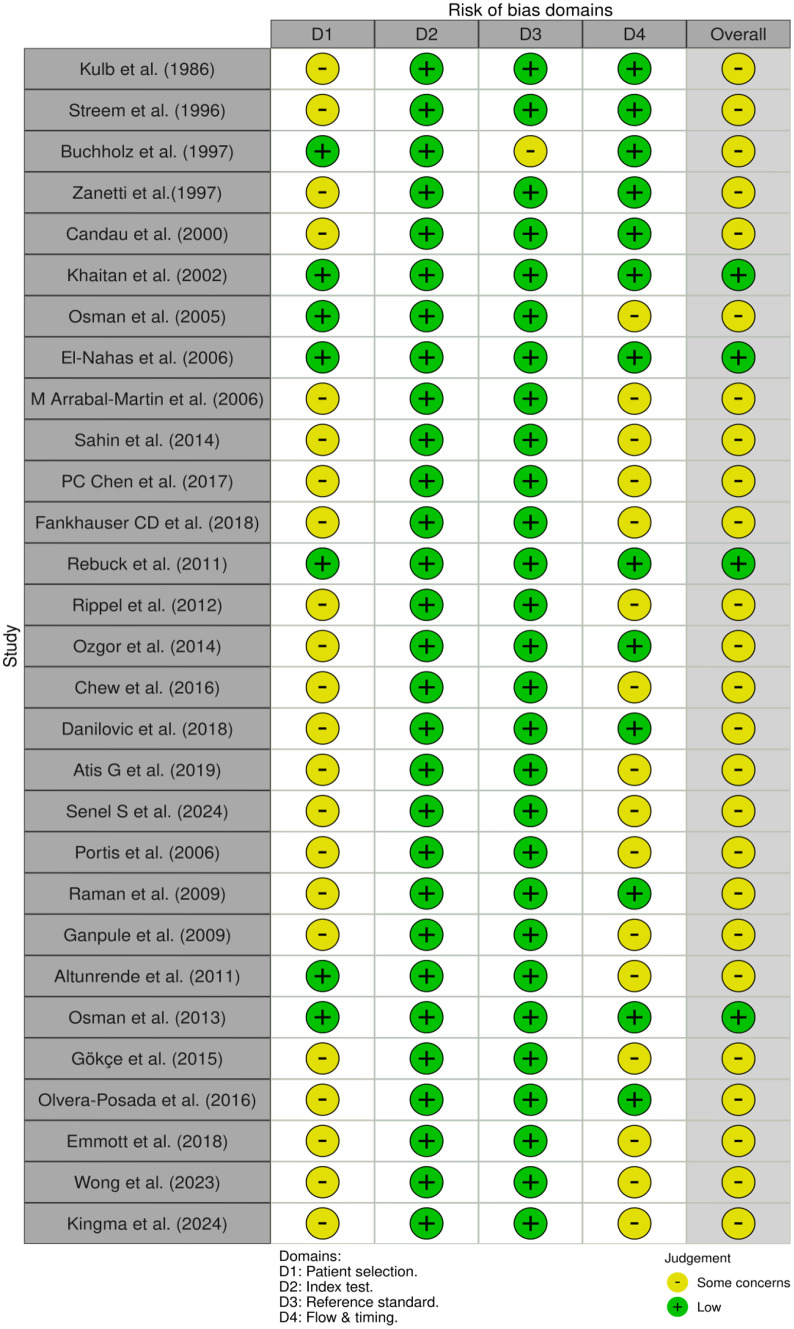
Risk of bias assessment with QUADAS-2 tool

**Fig. 3:**
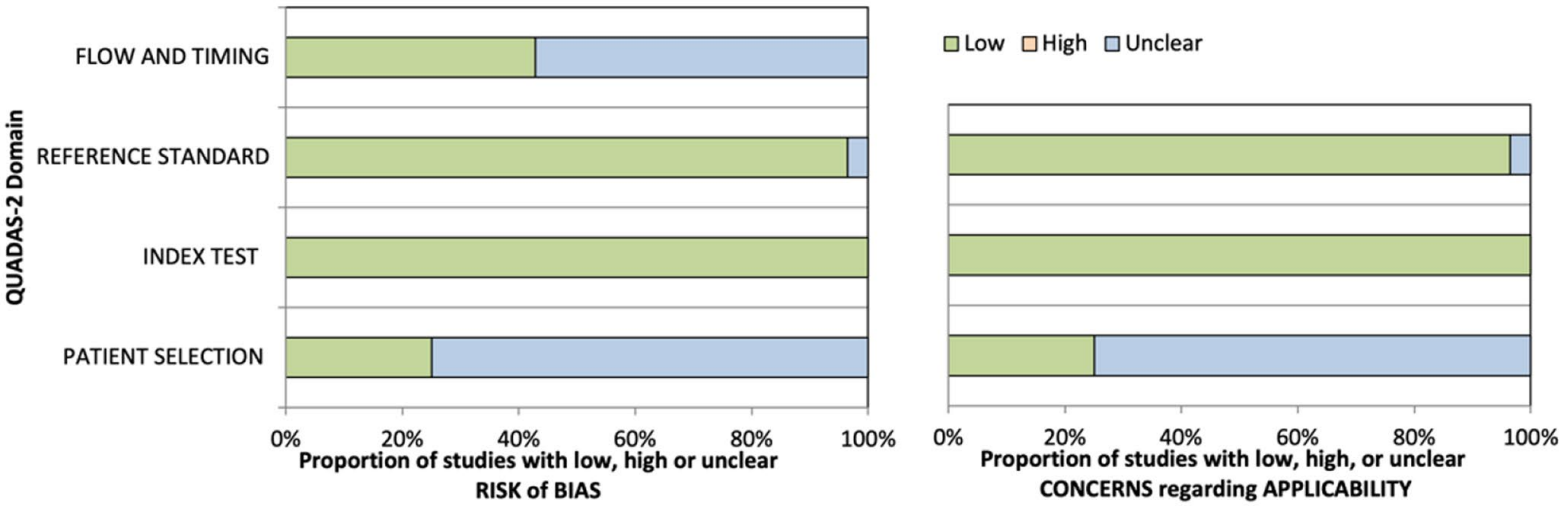
The proportion of studies regarding the risk of bias and concerns regarding applicability

Study qualities were assessed independently by two review authors (O.F.C., A.T.) using the Quadas scoring system [22] [Figs. 2 and 3] to identify risk of bias and concerns about applicability.

### Statistical analysis

Statistical meta-analysis was performed using the SPSS 29 with Meta-Analysis software (Chicago, USA). Heterogeneity was statistically calculated using the standard I2 test. In the present meta-analysis, I2 > 50% and a significance level of *p* < 0.10 for Cochran’s Q was considered as clinically important heterogeneity. Forest plot was constructed using a random or fixed model based on heterogeneity for RSF size, the only parameter whose standard deviation data could be measured. Furthermore, a funnel plot was generated to assess publication bias.

Pearson Correlation and linear regression analysis were performed for RSF%, spontaneous passage and intervention parameters. p value below 0.05 was considered statistically significant.

## Results

### Definition of RSFs

The nine studies (four ESWL [3, 23–25], one RIRS [8], four PCNL [9, 26–28]) defined it as RSFs < 4 mm, eleven studies (five ESWL [10, 29–32], three RIRS [11, 12, 33], three PCNL [13–15]) ≤ 4 mm, five studies (two ESWL [7, 16], one RIRS [34], two PCNL [17, 18]) < 5 mm and three studies (one ESWL [19], two RIRS [20, 21]) ≤ 2 mm while one study (PCNL [35]) as an area smaller than 25 mm² on X-ray. Tables 1 and 2, and 3 present the primary studies focusing studies on RSFs.

**Table 1:** Summary of the ESWL studies

Author/Year	Study type	Included patients	Where was definition made?	Composition of RF	BMI	Laser type/setting	Access sheathh	Total patients	Size of RSF	Number of RSF	Location of RSF	Presence of symptoms	Radio-opacity	HU of RSF	Diagnostic tool	Imaging time for RSF	SF status
Kulb et al.^16^/1986	Retrospective observational study	All solitary kidney patients, post ESWL	Policlinic	N/S	N/S	N/S	N/S	59	≤ 4 mm	N/S	N/S	No	N/S	N/S	X-ray and US	POD 1 and 3 months	RSF, 34% of all patients
Streem et al.^8^/1996	Prospective observational study	RSF < 4 mm, Asymptomatic	Policlinic	Calcium oxalatdphosphate	N/S	Dornier HM-3lithotriptor	N/S	160	< 4 mm	N/S	N/S	N/S	N/S	N/S	X-ray, IVU, or US	1 month	24% became stone-free
Buchholz et al.^26^/1997	Retrospective observational study	All patients post ESWL	Policlinic	Sterile purely CaOx stones	N/S	Piezolith 2300	N/S	44	< 5 mm	N/S	Lower calyx	No	Radiopaque	N/S	X-ray	1 month	At 1 month, RSF = 21%, After 2.5 years, spontaneous clearance in 88% of RSF, Especially located in the lower calyx are determinants of permanent RSF.
Zanetti et al.^36^/1997	Retrospective observational study	RSF ≤ 4 mm	Policlinic	Calcium oxalate and calcium phosphate	N/S	Dornier HM3 modified and MPL 9000 lithotriptor	N/S	129	≤ 4 mm	N/S	Lower calyx often	Yes	Radiopaque	N/S	X-ray and US	3,12,24,26 months	At the 12-month followup 46.5% were stone-free, regrowth rate was 13%
Candau et al.^9^/2000	Retrospective observational study	RSF ≤ 4 mm, noninfectious	Policlinic	N/S	N/S	Technomed sonolith 3000 lithotriptor	N/S	83	< 4 mm	N/S	Lower calyx (%62)	Yes	N/S	N/S	CT or IVU	3 months	RSF outcomes: 33% Passage, 29% decreased RF, 29% stable, 37% increased. 22% recommended re-intervention
Khaitan et al.^3^/2002	Prospective observational study	RSF ≤ 4 mm Nonobstructive, noninfectious, asymptomatic	Policlinic	N/S	N/S	Siemen’s Lithostar Plus	N/S	75	< 4 mm	2–3	%20 lower calyx, %4 pelvis	Yes	N/S	N/S	X-ray, subsequent IVU or US	3 months	RSFoutcomes: 24% passage, 18% stable, 59% regrowth, 31% re-intervention
Osman et al.^10^/2005	Prospective cohort study	RSF ≤ 4 mm Nonobstructive, noninfectious, asymptomatic	Policlinic	N/S	N/S	Modulith SLX or Modulith SLK electromagnetic lithotripters	N/S	173	< 4 mm	N/S	%16.8 lower calyx, %13.9 pelvis	N/S	N/S	N/S	IVU, Retrograde pyelography & US	N/S	79% RSF passed– most within 4 weeks, the proportion of ancillary treatments after ESWL was 44%
El-Nahas et al.^7^/2006	Retrospective cohort study	Asymptomatic RSF ≤ 5 mm	Policlinic	Calcium-containing stone	N/S	Dornier Lithotriptor S	N/S	154	< 5 mm	Single:%46 Multiple:%62	Lower calyx	N/S	N/S	N/S	CT	3 months	RSF: 14% passage, 53% stable, 34% re-growth
Arrabal-Martin et al.^17^/2006	Prospective cohort study	N/S	Policlinic	Calcium lithiasis	N/S	N/S	N/S	100	≤ 4 mm	N/S	Lower calyx %60	N/S	N/S	N/S	X-ray, US	N/S	RSFs was 28%, with clinical progression in 58% of patients and a stable situation in 18% of patients.
Sahin et al.^18^/2014	Prospective cohort study	Any RSF	Policlinic and emergency department	Calcium oxalate monohydrate in 13 (25%), calcium oxalate dihydrate in 5 (10%), and mixed calcium stones in 32 (65%; mixed with hydroxylapatite	N/S	Electromagnetic lithotripter	N/S	71	≤ 4 mm	2	N/S	Yes	N/S	690,00 ± 146,85	X-ray & US, with subsequent CT (KUB)	N/S	RSF, 59% < 4 mm 19% of RSF required medical management for symptoms, 21% reintervention
Chen et al.^19^/2017	Retrospective observational study	Solitary urinary calculus	Policlinic	N/S	26.3 ± 4.1	Dornier; DoLiS lithotriptor	N/S	641	≤ 4 mm	N/S	N/S	N/S	N/S	N/S	KUB	N/S	SFR, 46.7% for renal stones
Fankhauser et al.^29^/2018	Retrospective comparative cohort study	N/S	Policlinic	N/S	26.74 (4.75)	Dornier DL50 lithotripter	N/S	999	≤ 2 mm	Single	Lower calyx	N/S	N/S	N/S	KUB, US, CT	N/S	RSF, %29

Abbreviations: computed tomography (CT), extracorporeal shock wave lithotripsy (ESWL), intravenous urethrography (IVU), kidney-ureter-bladder (KUB), not specified (N/S), postoperative day (POD), residual stone fragments (RSF), stone-free (SF), ultrasonography (US)

**Table 2:** Summary of the RIRS studies

Author	Study type	Included patients	Where was definition made?	Composition of RF	BMI	Total patients	Laser type/setting	Access sheath	Surgeon experience	Size of RSF	Number of RSF	Location of RSF	Presence of symptoms	HU of RSF	Diagnostic tool	Imaging time for RSF	SF status
Rebuck et al.^11^/2011	Retrospective cohort study	RSF < 4 mm, non-cystine and non-struvite	Policlinic	calcium- based (%,90.2)	N/S	46	200-m fiber and holmium: YAG laser.	Yes(When feasible)	Single surgeon	< 4 mm	N/S	Lower calyx: %81,5	No	N/S	NCCT	3 months	RSF, 20% Interventional treatment in 13%, spontaneous passage in 22%, asymptomatic cases with no passage in 59%
Rippel et al.^30^/2012	Retrospective observational study	All patients post RIRS	Policlinic	Calcium oxalate monohydrate, Calcium phosphate, mixt	31	248	N/S	Yes(%49)	N/S	≤ 2 mm	N/S	N/S	N/S	N/S	NCCT	1–3 months	All procedures, RSF = 5%, No follow up data on outcomes
Ozgor et al.^6^/2014	Retrospective cohort study	RSF < 5 mm, asymptomatic, no obstruction or infection	Policlinic	Calcium oxalate, Calcium phosphate, Cysteine	25.3 + 3.9	44	N/S	Yes	N/S	< 5 mm	1–2	Lower calyx	Yes	N/S	NCCT	3 days-3 months	RSF, 34% symptomatic, 25% intervention, 11% stone growth
Chew et al.^20^/2016	Retrospective cohort study	RSF of any size	Policlinic and emergency department	N/S	N/S	232	N/S	N/S	N/S	≤ 4 mm	N/S	Lower calyx	N/S	N/S	X-ray (± US), or NCCT	Within 12 months	RSF, 44% had stone event, 29% intervention, 15% complication without reintervention
Danilovic et al.^31^/2018	Prospective observational study	N/S	Policlinic and intraoperative	N/S	N/S	115	270 micron Holmium laser fiber	Yes	Same surgeon	≤ 2 mm	2	Lower calyx	Yes	978.4 ± 333.1	NCCT	3 months	0–2 mm in 8.7% (10/115), and > 2 mm residual fragments in 16.5% (19/115) renal units
Atis et al.^21^/2019	Retrospective observational study	N/S	Policlinic	Ca oxalate/phosphate (32.55%),Uric acid (9.30%), Mixed (13.95%),Cystine (6.97%),Struvite (6.97)	N/S	142	200–273 μm laser fiber	Yes	N/S	≤ 4 mm	Single	N/S	N/S	N/S	CT	12 months	RSF, 60.5%; Spontaneous passing 30.23%
Senel et al.^22^/2024	Prospective observational study	RSF following RIRS	Policlinic	Mixed type	27.3 ± 4.5 kg/m2	115	Holmium-yttrium–aluminum-garnet laser (200–365 μm)	Yes	N/S	≤ 4 mm	N/S	Lower calyx	Yes	930,1 ± 318,5	CT	1–6 months	Spontaneous passing %26. 47.6% patients experienced stone re-growth

Abbreviations: computed tomography (CT), non-contrast-enhanced computed tomography (NCCT), not specified (N/S), residual stone fragments (RSF), retrograde intrarenal surgery (RIRS), stone-free (SF), ultrasonography (US)

**Table 3:** Summary of the PCNL of studies

Author/Year	Study type	Included patients	Where was definition made?	Composition of RF	BMI	Laser type/setting	Total patients	Surgeon experience	Size of RSF	Number of RSF	Location of RSF	Presence of symptoms	Radiological visibility	HU of RSFh	Diagnostic tool	Imaging time for RSF	SF status
Portis et al.^12^/2009	Prospective interventional study	Stone free or RSF of any size	Policlinic	Calcium oxalate%50, calcium phosphate%20, pure uric acid%25, mixed	29.2 kg/m2 .	Ultrasonic lithotripsy	25	Single surgeon	< 4 mm	N/S	N/S	N/S	Radiopaque	N/S	CT	24 h	RSF, 28% Intraoperative High magnification Rotational Fluoroscopy able to identify additional rf
Raman et al.^27^/2009	Retrospective cohort study	Any RSF	Policlinic	48% calcium oxalate monohydrate, 26% hydroxyapatite, 12% brushite, 10% calcium oxalate dihydrate and 5% cystine	N/S	N/S	42	N/S	< 5 mm	N/S	N/S	Renal pelvis or ureter may cause problems in the future	N/S	N/S	CT	1–21 days	RF, 43% stone related events, 26% re-intervention RSF > 2 mm or location in renal pelvis or ureter may cause problems in the future
Ganpule et al.^32^/2009	Retrospective observational study	Any RSF	Policlinic	N/S	N/S	N/S	187	More than 10 years of experience: A, fewer than 10 years of experience.:B	Area on X-ray < 25mm2	N/S	Lower calyx	N/S	N/S	N/S	X-ray & US	48 h & 1 month & 3 months	RSFs were identified in 7.57% of patients, RSF < 25mm2 and renal pelvis location = highest chance of passing
Altunrende et al.^13^/2011	Retrospective cohort study	RSF < 4 mm, asymptomatic, non-infectious	Policlinic	Calcium oxalate monohydrate in 81%, uric acid in 5%, and struvite stone in 13%	N/S	pneumatic lithotripter.	38	N/S	< 4 mm	N/S	Lower calyx % 60	N/S	N/S	N/S	X-Ray ± NCCT	48 h & 3 months	No association between RSF size & regrowth, All PCNL, 22% RSF at 3 months
Osman et al.^28^/2013	Retrospective observational study	RSF ≤ 5 mm, no infection	Policlinic	N/S	Average (30 kg/m2 )(43.9) Obese (30 kg/m2) (29.4)	N/S	75	N/S	< 5 mm	N/S	Lower calyx %38	Yes	Radiopaque %37.5	N/S	NCCT	N/S	29.35% of cases had stable fragments, 33.3% showed regrowth and in 4%, the fragment slipped into the ureter.
Gökçe et al.^14^/2015	Retrospective observational study	N/S	Policlinic	N/S	N/S	N/S	173	N/S	< 4 mm	N/S	N/S	N/S	N/S	N/S	NCCT, KUB, US	24–48 h	RSF, %24.3
Olvera-Posada et al.^23^/2016	Retrospective cohort study	RSF of any size	Policlinic and emergency department	N/S	30.6	N/S	44	N/S	≤ 4 mm	N/S	Lower calyx %61	Yes	N/S	N/S	NCCT	24–48 h	RSF %20 and 72.7% needed intervention
Emmott et al.^24^/2018	Retrospective cohort study	RSF of any size	Policlinic	N/S	24.3%	N/S	263	N/S	≤ 4 mm	Multiple	Lower calyx %52	N/S	N/S	N/S	NCCT	1 day	PCNL pts: 45% had RF ≥ 1 mm, Reintervention and regrowth: higher in > 4 mm (28%) vs. ≤ 4 mm (17%)
Wong et al.^15^/2023	Retrospective cohort study	RSF > 1 mm	Policlinic	N/S	31.7%	ultrasonic, mechanical, or laser lithotrites	439	Eight surgeons	< 4 mm	Multiple	Lower calyx %47	Yes	N/S	N/S	NCCT	1 day	RSF, %60 < 4 mm, RSF ≤ 2 mm had significantly higher rates of passage and lower rates of regrowth, complications, reintervention
Kingma et al.^25^/2024	Retrospective cohort study	N/S	Policlinic and emergency department	Calcium oxalate monohydrate;%38, Phosphate;%35, Uric acid;%14	27.1	N/S	103	N/S	≤ 4 mm	N/S	N/S	N/S	N/S	N/S	NCCT	N/S	RSF, %29

Abbreviations: computed tomography (CT), kidney-ureter-bladder (KUB), non-contrast-enhanced computed tomography (NCCT), not specified (N/S), residual stone fragments (RSF), stone-free (SF), ultrasonography (US)

### Patient groups

The studies evaluating ESWL, RIRS, and PCNL have grouped patients based on their inclusion criteria. For ESWL, one study included patients with solitary kidneys [29], two studies with non-obstructive, non-infectious, asymptomatic RSFs ≤ 4 mm [3, 25]. Some studies also included patients with asymptomatic RSFs ≤ 5 mm or solitary urinary calculi, irrespective of infection or obstruction status [7, 10]. RIRS studies predominantly included patients with RSFs < 4–5 mm, asymptomatic cases, and those with non-cystine and non-struvite residual stones [8, 34]. In contrast, others included patients with residual fragments of any size following RIRS [11, 12, 20]. For PCNL, inclusion criteria varied from stone-free status to the presence of residual fragments of any size [13, 14, 17, 26, 35], with a subset of studies focusing on asymptomatic, non-infectious RSFs ≤ 4–5 mm [18, 27].

### Diagnosis of RSFs

The most frequently utilized imaging technique for diagnosing RSFs was CT. Fourteen studies (one ESWL [7], six RIRS [8, 12, 20, 21, 33, 34], and seven PCNL [9, 13–15, 17, 18, 26]) used CT alone. Other studies have used a combination of imaging methods such as KUB graphs, US, intravenous ureterography (IVU), and X-ray to visualize RSFs.

### Clinical implications of RFs

We summarized the incidence, outcomes, and associated complications of RSFs using the following three common modalities: ESWL, RIRS, and PCNL.

### ESWL [Table 1

The incidence of RSFs following ESWL has been reported between 21% and 59% across the studies [3, 7, 10, 16, 19, 23–25, 29–31]. Kulb et al. [29] reported an RSF rate of 34% in solitary kidney patients after ESWL at 3 months. Streem et al. [23] found that 24% of patients with RSFs < 4 mm became stone free at 1 month. Buchholz et al. [16] observed a 21% RSF rate at 1 month, with 88% achieving spontaneous clearance within 2.5 years, particularly for lower calyx fragments. Arrabal-Martin et al. [30] showed an RSF rate of 28%, with 58% showing clinical progression and 18% remaining stable. Sahin et al. [31] reported that RSFs occurred in 59% of patients ≤ 4 mm. Chen et al. [10] documented a stone-free rate (SFR) of 46.7% for renal stones ≤ 4 mm. Fankhauser et al. [19] observed a RSFs rate of 29% for RSFs ≤ 2 mm.

Spontaneous stone passages of RSFs varied between 24% and 79%, with most fragments resolving within four weeks and were influenced by size and location [3, 7, 24, 25]. Candau et al. [24] reported a 33% passage rate for RSFs ≤ 4 mm at 3 months. Khaitan et al. [3] noted a 24% passage rate within 3 months. Osman et al. [25] observed a 79% passage rate for RSFs ≤ 4 mm, with most fragments passing within 4 weeks. El-Nahas et al. [7] showed a 14% passage rate for RSFs ≤ 5 mm at 3 months.

The reintervention rate for RSFs varied between studies. Candau et al. [24] found that 37% of RSFs increased in size, 22% required reintervention, while 29% decreased and 29% remained stable. Khaitan et al. [3] reported a regrowth rate of 59%, with 31% of cases requiring reintervention within 3 months, while 18% remained stable. El-Nahas et al. [7] observed a regrowth rate of 34% for RSFs at 3 months, and 53% remained stable. Sahin et al. [31] noted that 19% of RSF cases required medical management for symptoms, and 21% underwent reintervention. Osman et al. [25] reported that 44% of RSFs required additional treatments.

A significant proportion of the evidence indicates that following ESWL, most RSFs are located in the lower pole. This anatomic location is associated with the lowest clearance rate and the highest risk of stone-related events. Regarding fragment size, fragments > 4 mm had increased complications, regrowth, and reintervention rates [3, 7, 16, 19, 24, 30].

### RIRS [Table 2

Among the RIRS studies, RSF rates varied between 20 and 60.5% of patients [8, 11, 12, 20, 21, 33, 34]. Rebuck et al. [8] reported an RSF rate of 20%, with 13% of patients requiring intervention, 22% achieving spontaneous passage, and 59% remaining asymptomatic without passage at three months. Rippel et al. [20] found an RSF rate of 5% after RIRS, but no follow-up data on outcomes were available. Ozgor et al. [34] observed a 34% RSF rate, with 25% requiring intervention, 11% experiencing stone regrowth, and all cases remaining asymptomatic during follow-up (3 days to 3 months). Chew et al. [11] reported an RSF rate of 44%, with 29% undergone intervention and 15% experiencing complications without reintervention within 12 months. Danilovic et al. [21] recorded RSF rates of 8.7% for fragments 0–2 mm and 16.5% for fragments > 2 mm at three months. Atis et al. [33] also noted a 60.5% RSF rate.

Spontaneous passage rates were affected by fragment size, location, and follow-up periods. Spontaneous passage of fragments occurred in 8.7–30.23% of cases, while a significant proportion of patients (59%) remained asymptomatic without fragment passage [8, 12, 21, 33]. Rebuck et al. [8] reported a 22% spontaneous passage rate for RSFs ≤ 4 mm within three months. Danilovic et al. [21] found an 8.7% passage rate for RSFs 0–2 mm, indicating that smaller fragments were more likely to pass. Atis et al. [33] reported a 30.23% spontaneous passage rate within 12 months. Similarly, Senel et al. [12] noted a 26% spontaneous passage rate for RSFs ≤ 4 mm over six months.

The rates of regrowth and reintervention varied between studies. However, interventions were required in 13–29% of cases due to symptomatic RSFs [8, 11, 34], stone regrowth was reported in 11–47.6% of patients, and complications occurred in 15% of cases, even without reintervention [11, 12, 34]. In addition, stone-related events were documented in 44% of cases, with intervention rates reaching as high as 25% [11, 34]. Rebuck et al. [8] reported that 13% of cases required intervention, although no specific data on regrowth were noted. Ozgor et al. [34] found that 25% of RSF cases required intervention, with 11% showing stone regrowth within the follow-up period. Chew et al. [11] indicated that 29% of cases required intervention, while an additional 15% experienced complications without reintervention. Senel et al. [12] reported a high regrowth rate of 47.6% within 1–6 months, highlighting the need for careful monitoring after RIRS.

The lower calyx was the predominant location for RSFs across the studies [8, 11, 12, 34]. Danilovic et al. [21] found that RSFs 0–2 mm and > 2 mm were predominantly in the lower calyx.

### PCNL [Table 3

RSFs were observed between 20 and 60% after PCNL [13, 15, 17, 26–28, 35]. Portis et al. [26] reported an RSF rate of 28%, which was identified using intraoperative high-magnification fluoroscopy. Altunrende et al. [27] observed an RSF rate of 22% at three months. Gökçe et al. [28] showed a 24.3% RSF rate after PCNL. Olvera-Posada et al. [13] identified RSFs in 20% of patients, with 72.7% requiring intervention. Emmott et al. [14] found that 45% of PCNL patients had RSFs ≥ 1 mm, and Wong et al. [9] reported that 60% had RSFs ≤ 4 mm, with higher passage rates in fragments ≤ 2 mm. Kingma et al. [15] observed an RSF rate of 29% for fragments ≤ 4 mm.

Spontaneous passage rates were closely linked to fragment size and location. Ganpule et al. [35] found that fragments < 25 mm² in the renal pelvis had the highest passage chances. Wong et al. [9] reported significantly higher passage rates for fragments ≤ 2 mm than larger fragments, highlighting size as a critical determinant of spontaneous passage.

Regrowth was reported in several studies, varying with varying rates depending on fragment size and follow-up timing. Altunrende et al. [27] found no significant association between RSF size and regrowth. Osman et al. [18] found 29.35% of cases had stable fragments, with 33.3% showing regrowth and 4% fragment migration to the ureter. In comparison, Emmott et al. [14] reported higher regrowth and reintervention rates for fragments > 4 mm (28%) compared to ≤ 4 mm (17%).

Reintervention rates were highest in studies where larger fragments (> 2 mm) or locations, such as the renal pelvis or ureter, were present [9, 13, 14, 17, 18, 27, 35]. Raman et al. [17] noted a 26% reintervention rate, with fragments > 2 mm posing future complications. Similarly, Olvera-Posada et al. [13] found 72.7% of RSF patients required reintervention.

In most studies, the lower calyx was the most common location for RSFs, reported in 38–61% of cases. Most studies consistently identified the lower calyx as a prominent RSF location [9, 13, 14, 18, 27, 35]. However, two studies emphasized that fragments in the renal pelvis or ureter, although less frequent, had higher risks of complications and lower chances of spontaneous passage, making their management more critical [17, 35].

### Statistical analysis results

**Fig. 4:**
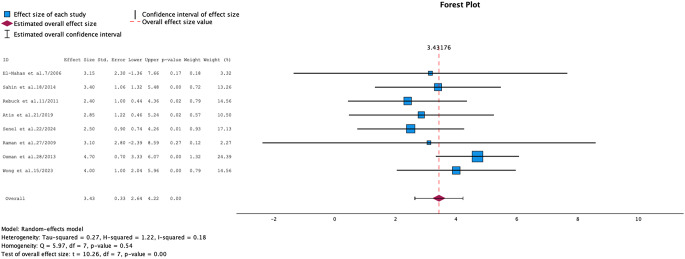
Forest plot for heterogeneity analysis

In the heterogeneity analysis performed according to RSF size + SD values, it was determined that the studies were low heterogeneous. The forest plot graphic of the studies is observed in Fig. 4. (95% CI: [2.59; 4.27], *p* = 0.54, I² = 18%, τ² = 0.27)

**Fig. 5:**
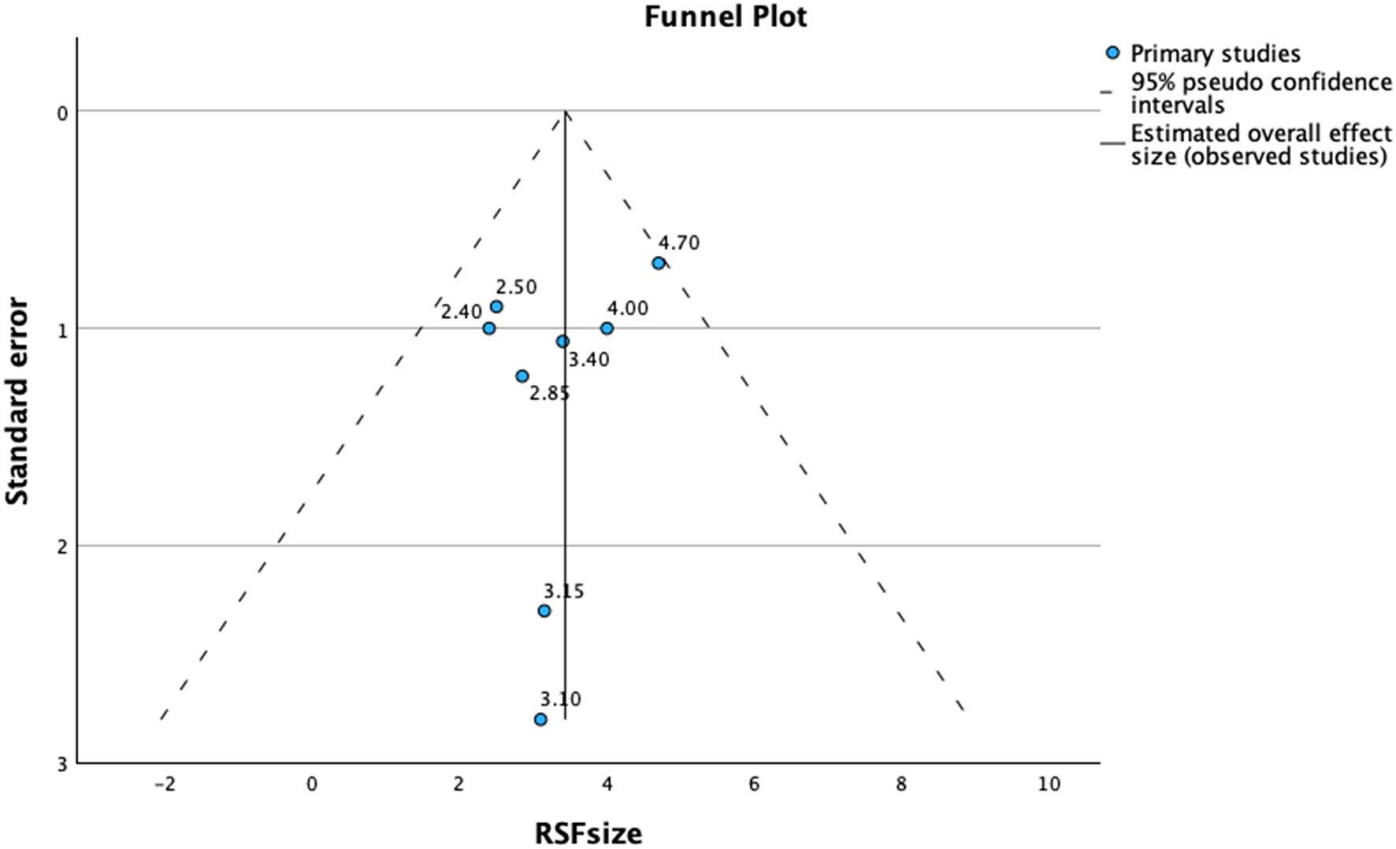
Publication bias graph

Publication bias; There was no evidence of publication bias in favor of studies reporting a RFS size using Egger’s test (intercept = 0.004, t value = -0.89, 2-tailed *p* = 0.40) (Fig. 5).

**Fig. 6:**
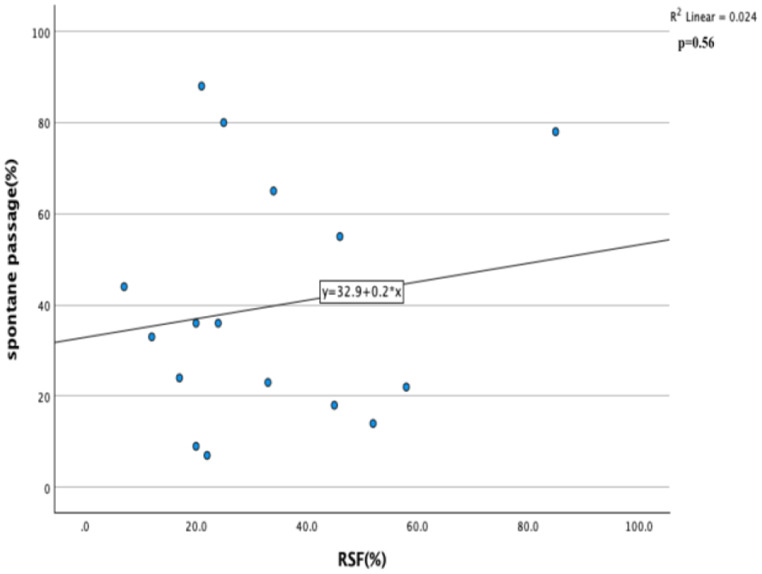
Correlation and linear regression analysis of spontane passage

**Fig. 7:**
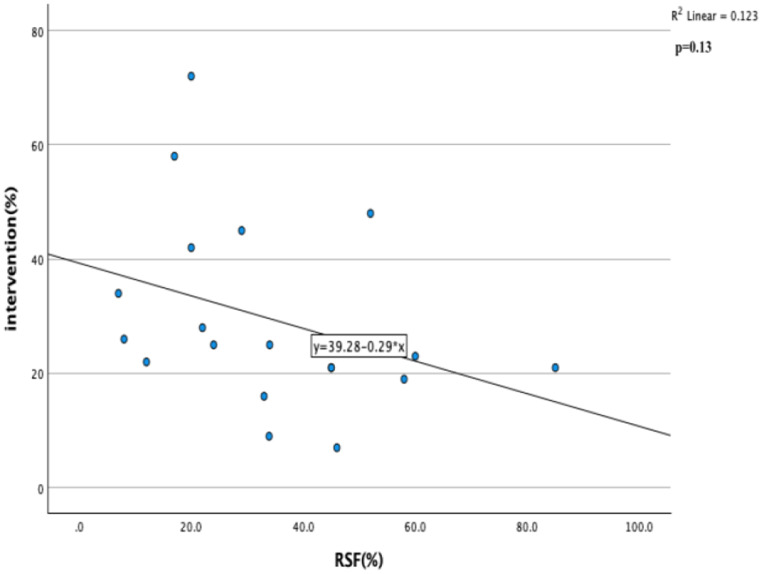
Correlation and linear regression analysis of intervention

Correlation and linear regression analysis were performed for RSF%, spontaneous passage and intervention parameters. A positive correlation was found between RSF% and spontaneous passage (Fig. 6) and a negative correlation was found between RSF% and intervention (Fig. 7), but this relationship was not statistically significant (r: 0.15, p: 0.56, r: -0.35, 0.13 respectively). Although not significant, the relationship between these parameters was interpreted as clinically insignificant stones that can be passed with spontaneous passage and require less intervention are given more attention.

### Suggested Follow-up strategy for residual stone fragments

Based on the proposed risk stratification, a structured follow-up strategy is recommended (Table 4):

- Low-risk RSFs (≤ 4 mm, asymptomatic, no metabolic risk): Follow-up with US/KUB at 6–12 months. If stable or resolved, follow-up can be shaped based on individual patient conditions. If growth occurs, reassess the risk level.
- Intermediate-risk RSFs (≤ 4 mm, occasional symptoms, or metabolic risk): US/KUB every 6–12 months. If the fragment persists or symptoms worsen, earlier imaging and reintervention should be considered.
- High-risk RSFs (> 4 mm, symptomatic or asymptomatic, with or without metabolic risk): Early reintervention should be considered. If a conservative approach is chosen, US/KUB at 3–6 months is recommended. If the fragment grows, symptoms develop, or new stones appear, CT and surgical intervention should be performed.

**Table 4:** A structured risk stratification and follow-up strategy for residual stone fragments

Risk Category	Follow-up Strategy
Low-risk RSFs (≤ 4 mm, asymptomatic, no metabolic risk)	US/KUB at 6–12 months → If stable or resolved, follow-up can be shaped based on individual patient conditions. If growth occurs, reassess the risk level.
Intermediate-risk RSFs (≤ 4 mm, occasional symptoms, or metabolic risk)	US/KUB every 6–12 months → If the fragment persists or symptoms worsen, earlier imaging and reintervention should be considered.
High-risk RSFs (> 4 mm, symptomatic or asymptomatic, with or without metabolic risk)	Early reintervention should be considered → If a conservative approach is chosen, US/KUB at 3–6 months is recommended. If the fragment grows, symptoms develop, or new stones appear, CT and surgical intervention should be performed.

## Discussion

RSFs represent a critical challenge in urological practice following stone treatment procedures. This systematic review highlights the significant variability in RSF definitions and outcomes across treatment modalities, including ESWL, RIRS, and PCNL. The lack of standardized definitions for RSFs limits the comparability of studies and hinders the development of unified treatment guidelines. A recent systematic review aimed at developing a tracking algorithm determined the RSF cut-off value to be 4 mm. However, it is important to note that studies setting it at 2 mm and 5 mm could also be considered to provide realistic results [36]. The EAU guideline utilises a 4 mm cut-off value for residual fragment management; however, it is acknowledged that around a third of patients of smaller stones undergo disease progression and require re-intervention within three years [6]. While most studies focus on size, this review emphasizes the importance of additional parameters such as localization, and density in evaluating RSF behavior, recurrence risks, and reintervention requirements. Current evidence indicates that RSFs are not clinically insignificant, with significant implications for patient outcomes, particularly regarding regrowth, infection, and obstruction risks. The review underscores the necessity of optimizing procedural techniques, careful monitoring, and individualized treatment strategies to minimize RSF incidence. It also advocates for a systematic approach to standardizing RSF definitions by incorporating clinically significant parameters beyond size. This systematic review has demonstrated significant heterogeneity with regard to the monitoring method and the time of imaging following the intervention in question. By addressing these critical gaps, this review aims to improve the quality of research and inform clinical practices for better patient management.

The incidence of RSFs varies widely depending on the treatment method. For ESWL, RSFs have been reported in 21–59% of cases [3, 7, 10, 16, 19, 23–25, 29–31]. Among solitary kidney patients, Kulb et al. [29] reported an RSF rate of 34% at three months, while Sahin et al. [31] showed RSFs in 59% of patients with fragments ≤ 4 mm. RIRS shows similar variability, with RSF rates ranging from 20 to 60.5% [8, 11, 12, 20, 21, 33, 34]. For PCNL, the reported RSF incidence ranges from 20 to 60% [13, 15, 17, 26–28, 35], with Portis et al. [26] identifying RSFs in 28% of cases using high-magnification fluoroscopy. In light of these findings, it is undeniable that residual stone fragments are an inherent reality of urological practice, regardless of the treatment modality, and their prevalence is far from negligible.

The spontaneous passage of RSFs is highly dependent on fragment size and location. Smaller fragments (≤ 2 mm) have significantly higher clearance rates than larger fragments. Wong et al. [9] reported that fragments ≤ 2 mm were more likely to pass spontaneously. Similarly, Osman et al. [25] observed a 79% passage rate for RSFs ≤ 4 mm after ESWL, with most fragments clearing within four weeks. However, lower calyx fragments consistently demonstrate the lowest passage rates, likely due to unfavorable anatomical factors. This location-specific challenge is a recurring issue across all treatment modalities [8, 11, 12, 34].

RSF regrowth remains a critical concern, particularly for fragments > 4 mm. Studies have reported that regrowth rates could vary between 33% and 59% after ESWL and PCNL [3, 18, 24]. For RIRS, regrowth rates are similarly high, with Senel et al. [12] presenting regrowth in 47.6% of cases within six months. Reintervention rates are also substantial, reflecting the clinical burden of residual fragments. Olvera-Posada et al. [13] found that 72.7% of patients with RSFs after PCNL required reintervention. Chew et al. [11] reported that 29% of patients undergoing RIRS required additional interventions within 12 months. However, a systematic review assessing the significance of residual fragments identified 18 studies that demonstrated no significant difference in rates of re-intervention and disease progression, regardless of fragment size [37]. We can assume that there is a necessity for careful post-treatment monitoring and early intervention when regrowth or symptomatic fragments are identified.

The lower calyx is the most problematic location for RSFs across all treatment modalities. This location exhibits the lowest clearance rates and presents a higher risk for future complications. Studies consistently identify the lower calyx as the predominant site for RSF retention, making it a focal point for targeted therapeutic strategies [8, 11, 12, 34]. Conversely, fragments in the renal pelvis have shown higher spontaneous passage rates, as reported by Ganpule et al. [35]. This highlights the importance of considering the fragment location in clinical decision-making and patient counseling.

While the definitions of RSF are debatable, the term “stone DUSTING” i.e. conversion of stones to very small sand like particulate using higher frequencies, is becoming a common practice due to the emerging technologies. However, research in this area is evolving, with a few available in vivo and in vitro studies. Additionally, there is a lack of data on imaging modalities to assess “stone DUST” and its relationship with RSF [38, 39].

Recent technological advancements have led to significant progress in reducing RSF. A study conducted in 2024 by W. Zhu et al. [40] compared the utilisation of a flexible vacuum ureteral access sheath with a conventional sheath in RIRS procedures. The investigation revealed a reduced RSF rate with the utilisation of the vacuum ureteral access sheath.

The existing literature demonstrates an urgent need for a broader perspective on RSF evaluation, extending beyond size to include parameters like stone composition, localization, and density. These factors could provide a more comprehensive understanding of RSF behavior, recurrence risk, and the need for further interventions. A systematic review aimed at standardizing the definition of residual fragments, incorporating these additional dimensions, would fill a significant gap in the current body of research and could reshape clinical practices in urology. These findings critically impact patient management and highlight the importance of procedural optimization, careful monitoring, and individualized treatment strategies.

Lombardo et al. [36] proposed a classification system that primarily categorizes patients based on stone-free status and a strict size cutoff at 4 mm. While their classification provides a structured framework for follow-up, it lacks detailed symptom-based and metabolic considerations. In contrast, our proposed model integrates symptomatology, metabolic risk, and recurrence history to enhance clinical decision-making. Notably, our high-risk RSF category includes both symptomatic and asymptomatic patients > 4 mm, reflecting the variable clinical course of residual fragments. This refinement ensures more individualized follow-up strategies, bridging the gap between simplistic size-based classification and patient-specific risk assessment.

This systematic review shows that stone size has been considered in the literature as an essential determinant of the presence of RSF. However, other potential factors such as stone composition, patient body mass index (BMI), device type and settings, use of access sheaths and surgeon experience have not been comprehensively evaluated. Future studies incorporating all these factors are needed to predict the presence of significant RSF more accurately.

## Conclusion

Most studies define RSF as fragments < 4 mm, while thresholds of < 2 mm and < 5 mm were also common. Computed tomography (CT) is the most reliable diagnostic modality, particularly for identifying fragment size and their location. The review highlights that RSFs exceeding 4 mm, especially in the lower pole, are associated with increased risks of progression, reintervention, and complications. The study provides a solid foundation for establishing standardized definitions and diagnostic criteria to improve treatment planning, and patient outcomes.

## Data Availability

No datasets were generated or analysed during the current study.
